# Rapid surveillance of New York City healthcare center egress behaviors during the 2020 COVID-19 lockdown

**DOI:** 10.1038/s41597-023-02692-0

**Published:** 2023-11-11

**Authors:** Thomas Kirchner, Haoran Jiang, Hong Gao, Germaine Kabutaulaka, Darlene Cheong, Yungi Jiang, Aseah Khan, Weiyi Qiu, Nikki Tai, Tiffany Truong, Maimunah Virk, Peter Gmelch, Chris Carey, Debra Laefer

**Affiliations:** 1 Department of Social and Behavioral Sciences, School of Global Public Health, New York University, New York, NY USA; 2 Center for Data Science, New York University, New York, NY USA; 3 Department of Epidemiology, School of Global Public Health, New York University, New York, NY USA; 4 Department of Biology, New York University, New York, NY USA; 5 Tandon School of Engineering, New York University, New York, NY USA; 6 Courant Institute of Mathematical Sciences, New York University, New York, NY USA; 7 College of Arts and Science, New York University, New York, NY USA; 8 Center for Urban Science and Progress, Tandon School of Engineering, New York University, New York, NY USA

**Keywords:** Risk factors, Viral infection

## Abstract

This rapid response surveillance project was funded by the National Science Foundation (NSF) to collect “perishable” data on egress behaviors and neighborhood conditions surrounding healthcare centers (HCCs) in New York City (NYC) during the initial NYC COVID-19 PAUSE ordinance from March 22nd to May 19th, 2020. Anonymized data on NYC HCC egress behaviors were collected by observational field workers using phone-based mapping applications. Each egress trip record includes the day of week, time of day, destination category type, along with an array of behavioral outcome categories, ambient weather conditions and socio-economic factors. Egress trajectories with precise estimates of distance traveled and the spatial dispersion or “spread” around each HCC were added via post-processing. The data collection and cleaning process resulted in 5,030 individual egress records from 18 facilities over a 9-week period.

## Background & Summary

**Fig. 1:**
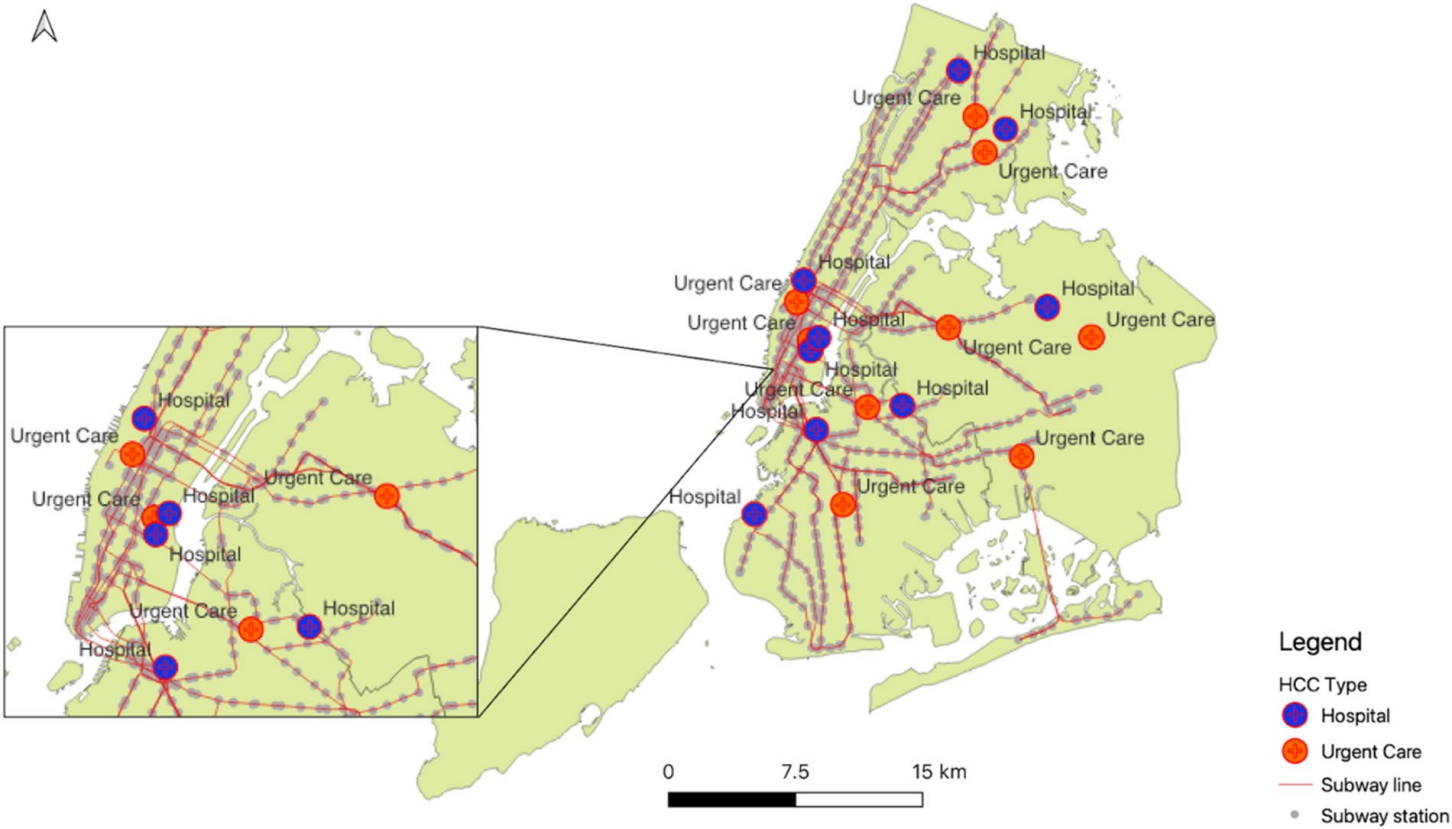
Healthcare Center (HCC) Types surveyed layered with subway routes.

During initial implementation of the COVID-19 PAUSE ordinance in New York City (NYC), enacted between March 22 – June 13, 2020 (https://www.governor.ny.gov/news/governor-cuomo-signs-new-york-state-pause-executive-order), there was a suspension of nonessential businesses. Yet transportation workers, police, and essential healthcare workers remained on the job and the employment scope of many required their presence at a healthcare center (HCC). The present project stems from this early phase of the pandemic, when the National Science Foundation funded an effort to collect “perishable” data on neighborhood conditions and egress behaviors around eighteen (18) NYC-based healthcare centers, broadly defined to include both larger hospital centers (N = 11) and local urgent care clinics (N = 7; Fig. 1). The NYC lock-down period afforded a useful opportunity to observe this subtle yet essential aspect of HCC utilization, as patients and staff exit and re-connect with the surrounding community.

This project aims to expand what is known about the vector environments surrounding HCCs, which are essential service points that remain open during administrative shut-down orders. Vector control strategies in public health involve efforts to contain or mitigate the spread of disease by intervening upon the vectors — such as modes of transportation — that carry disease agents to susceptible hosts^1^. When disease transmission accelerates into what is commonly called “viral” spread, as with COVID-19, members of the population serve as both susceptible hosts and as vectors for transmission of the disease agent to other people^2^. Governmental efforts to implement vector control measures restrict opportunities for people to engage with physical spaces and social environments, including “surface vector” fomites such as door handles, hand rails and clothing so that opportunities for disease spread through the population are minimized^3^.

Vector control strategies remain central to the mitigation and containment of COVID-19, but focus has thus far been limited to the operational status of non-essential retail, recreation and entertainment venues, with less attention on the hyper-local conditions surrounding neighborhood healthcare centers. This 9-week observational study established a protocol and archival dataset describing the egress behaviors of individuals leaving HCCs located in different areas of New York City during the 2020 COVID-19 PAUSE order. This paper introduces the dataset and presents a preliminary description of the social and geographic indicators available for each healthcare center. The project protocol was reviewed and deemed exempt by New York University’s institutional review board (IRB-FY2020-4305) prior to the initiation of field work.

## Methods

The data collection protocol was designed to capture anonymized, hyper-local data on HCC egress dynamics that would not be possible to ascertain from cellular data records, footfall data, closed-circuit television, or traffic cam data. In the most basic sense, egress behaviors were operationalized as the trajectory and total distance of the travel path taken by HCC visitors exiting each HCC and walking to their next destination. The a priori intent was to have a balanced distribution of observation sites between hospitals (i.e., including and emergency room; ER), and urgent care clinics across multiple NYC boroughs. To that end, a team of field observers was contracted to conduct 160 hours each of passive behavioral surveillance. However, the local conditions across NYC generated much higher foot traffic at some facilities than others.

### Field collection procedures: anonymized surveillance of healthcare center egress behaviors

Local resident fieldworkers (i.e., New York University students) were recruited to work as observers immediately prior to New York’s implementation of the PAUSE order at 8PM on March 22, 2020. The study was funded for 9 weeks with student observers collecting data up to. 20 hours per week until May 19, 2020. Ultimately, 18 facilities across 4 of New York City’s 5 boroughs (Queens, Brooklyn, Manhattan, and the Bronx) were selected based on the ability of the observer to reach the selected location by foot so that the observers would not have to undergo COVID-19 exposure in public transportation or for hire vehicles. When multiple facilities met this criterion, the one closest to a subway station was selected to capture the egress destination behavior with respect to public transportation.

Procedurally, field observers positioned themselves across the street from their assigned HCC egress location and then traced each subject’s egress route, noting locations of interactions with the built environment or other individuals. On Apple iPhones, the data were collected via DrawMaps, while on Android phones the MyMaps app was used. The egress route duration and time of day were recorded, as well as locations visited, and if applicable, transportation choices (e.g., bus, subway, Citibike, taxi, Uber, personal vehicle). Observations did *not* include photographs, video, or any interactions with the subjects, nor any other assessments of subjects’ identity or infection status, so as to preserve anonymity and minimize disturbances, as well as to protect the observer from exposure to COVID-19. As there was no guidance for mask usage at the onset of the study, collection of personal protective equipment (PPE) usage was not part of the formal data collection protocols, but over 1,800 of the more than 5,000 records do contain this information.

Final destinations were categorized by location type (e.g., coffee shop, pharmacy, deli, food trucks), including whether subjects returned to the medical facility or entering a nearby one (e.g., temporary tent, adjacent clinic or campus building). For each HCC, egress recordings extended from the same pre-specified point, until one of three outcomes occurred: (1) the subject entered a vehicle, subway station, building or other destination where the subject was no longer visible from the street, 2) tracking exceeded 20 minutes (an average observation period lasted 5 minutes in duration); or (3) the subject walked more than 1.3 km from the HCC, as a mile is considered the upperbound of a walkable distance in many urban planning contexts^4,5^ and was also an upperbound mobility restriction in other communities^6,7^.

Post-processing of the behavioral dataset also involved extraction and coding of meta-data – notes associated with each behavioral record and included in the attribute table associated with each shape file. Descriptive notes were extracted manually from the KML/KMZ files and entered into a spreadsheet. To introduce quality control measures, the notes were scraped by one researcher and checked on an entry-by-entry level by a second researcher. A secondary coding was also conducted on some key data fields, standardizing and allowing for a more generalized accounting of the data. For example, taxi, Uber, and Lyft were combined in the secondary coding as “vehicle for hire”.

### Calculation of egress trajectory distances and geographic dispersion around each HCC

A unique aspect of this data set is that it can be used evaluate and compare spatial dispersion around each HCC facility over time and between facilities. Spatial dispersion was defined as the spatial magnitude of the geographic area encompassing all egress trajectory records from each HCC and was approximated with a minor adaptation of the well-established radius of gyration (*R*_*g*_) statistic^8^, which is essentially the standard deviation of a set of locations around their center of mass, typically reported in meters. To facilitate research on the neighborhood areas around each HCC, we calculated a collective *radius of egress R*_*e*_ statistic that centers on each HCC exit point (rather than the center of mass used to calculate *R*_*g*_). This *R*_*e*_ metric provides a standardized estimate of the spatial dispersion associated with the egress records collected from each HCC facility,

$${R}_{e}=\sqrt{\frac{1}{n}\mathop{\sum }\limits_{k=1}^{n}{\left({d}_{k}-HC{C}_{exit}\right)}^{2}}$$

where *n* is the total number of egress records collected from each HCC, and *HCC*_*exit*_ is the location center of mass, or the longitude and latitude of each HCC exit point. The great circle distance in meters between the final destination observed for each egress record and their collective center of mass (*d*_*k*_-*HCC*_*exit*_) was calculated using Vincenty’s formulae^9^.

### Neighborhood estimates associated with each HCC

An array of socio-economic indicator variables used by the Center for Disease Control (CDC) to calculate the Social Vulnerability index (SVI) were included in the archived dataset, each linked to the geo-rectified location of the HCC entry/exit points used for field observations. The majority of variables included were taken from the American Community Survey (ACS; 2014–2018) with estimates of and margins of error provided in numbers and percentages for the total population, along with housing units, and other standard household indicators for the zip-code around each HCC. To enrich and ease re-use for future analysis, also included in the archival dataset were metadata and indicators of ambient weather conditions (on an hourly basis) outside each HCC on the days that data collection occurred.

## Data Records

**Fig. 2:**
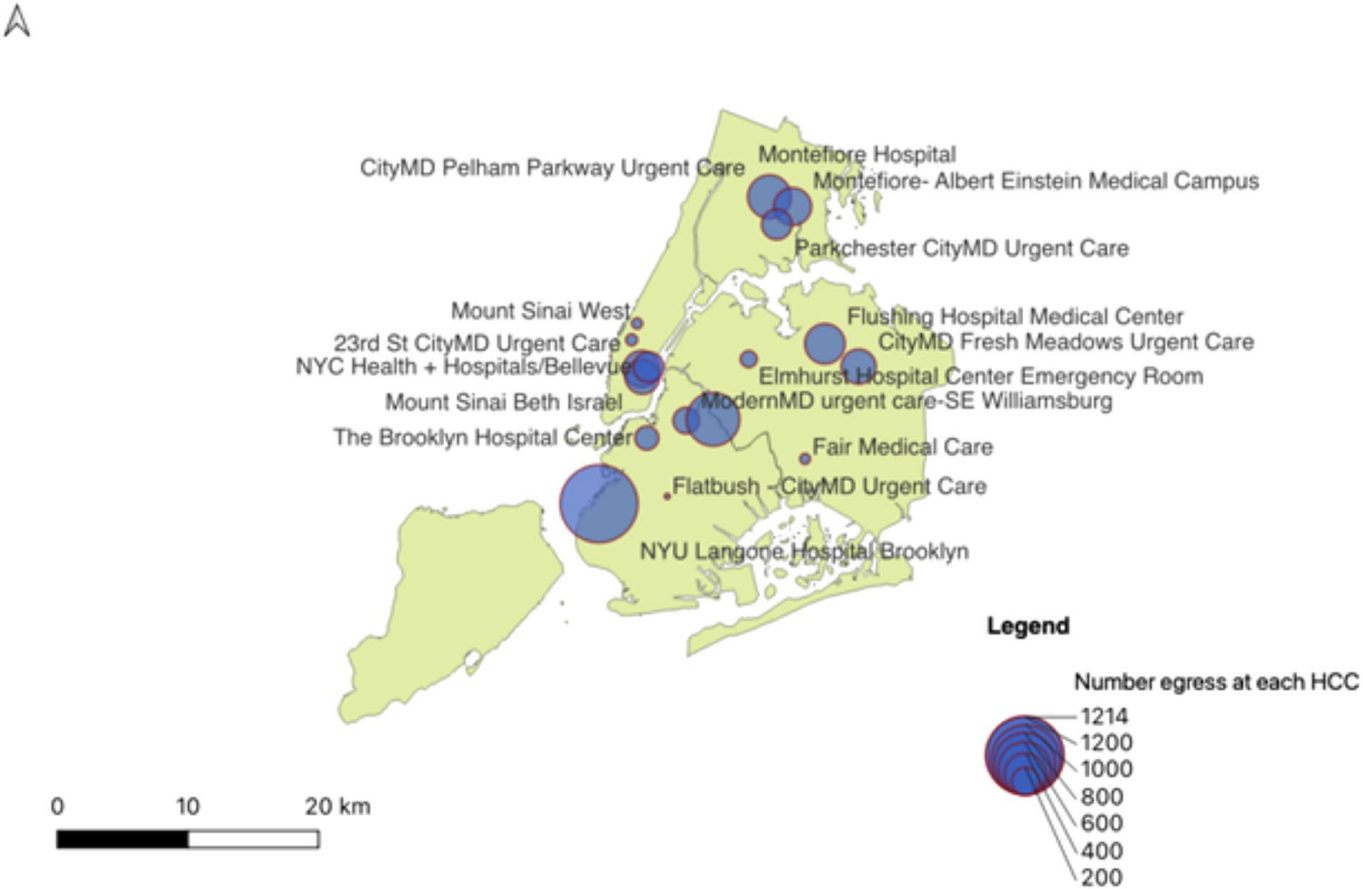
Number of subjects observed to egress each Healthcare Center.

All available data and the associated codebook are accessible through the NYU Faculty Digital Archive^10^. The codebook explains on a column-by-column basis the variables in the master CSV file, providing the variable format, naming conventions and labels, description, missing value indicators, and data storage type (e.g. binary, string, character, numeric). Altogether over 1,500 hours of raw data collected during daylight hours between March 22–May 19, 2020. Egress records associated with each HCC facility were merged into a unified geo-database. The data collection and cleaning process resulted in 5,030 individual egress records from 18 facilities, 11 of which were hospital centers and 7 that were urgent care centers (Fig. 2). The project produced a mean N = 280 egress records per HCC. There were 1,035 subjects observed in the Bronx, 2,210 in Brooklyn, 968 in Manhattan, and 822 subjects in Queens. The project’s intent was to collect a balanced data set across facilities and boroughs. To this end each observer was given the same number of weeks and hours per week. However, local infection rates heavily impacted footfall at each location. Furthermore, observer recruitment was opportunistic based on those who applied. Within that pool, the study tried to include a similar number of facilities per borough. No Staten Island based applications were received. Table 1 presents demographic statistics for each borough in the study, including population, age distribution, and education level.

**Table 1:** Demographic breakdown of each borough according to the 2014-2018 American Community Survey zip-code of facilities.

	Bronx	Brooklyn	Manhattan	Queens
Estimated population	1,437,872	2,600,747	1,632,480	2,298,513
Number of egresses observed (%)	1,035 (20.56)	2,210 (43.89)	968 (19.23)	822 (16.33)
Estimated number of minority residents (%)	1,304,150 (90.7)	1,659,277 (63.8)	866,847 (53.1)	1,716,989 (74.7)
Persons below the poverty level (%)	418,421 (29.1)	548,758 (21.1)	270,992 (16.6)	298,807 (13.0)
Estimated rate of unemployment	70,250	91,531	54,298	74,382
Estimated number of persons with no high school diploma (%)	258,956 (18.0)	326,469 (12.55)	162,237 (9.94)	303,881 (13.22)
Estimated number of persons aged 17 and younger	361,080 (25.11)	599,759 (23.06)	235,771 (14.44)	465,458 (20.25)
Estimated number of persons aged 65 and older	174,470 (12.13)	343,548 (13.21)	257,362 (15.77)	340,656 (14.82)

*All persons except white, non-Hispanic from 2014-2018 ACS for zip-code of facility.

**Fig. 3:**
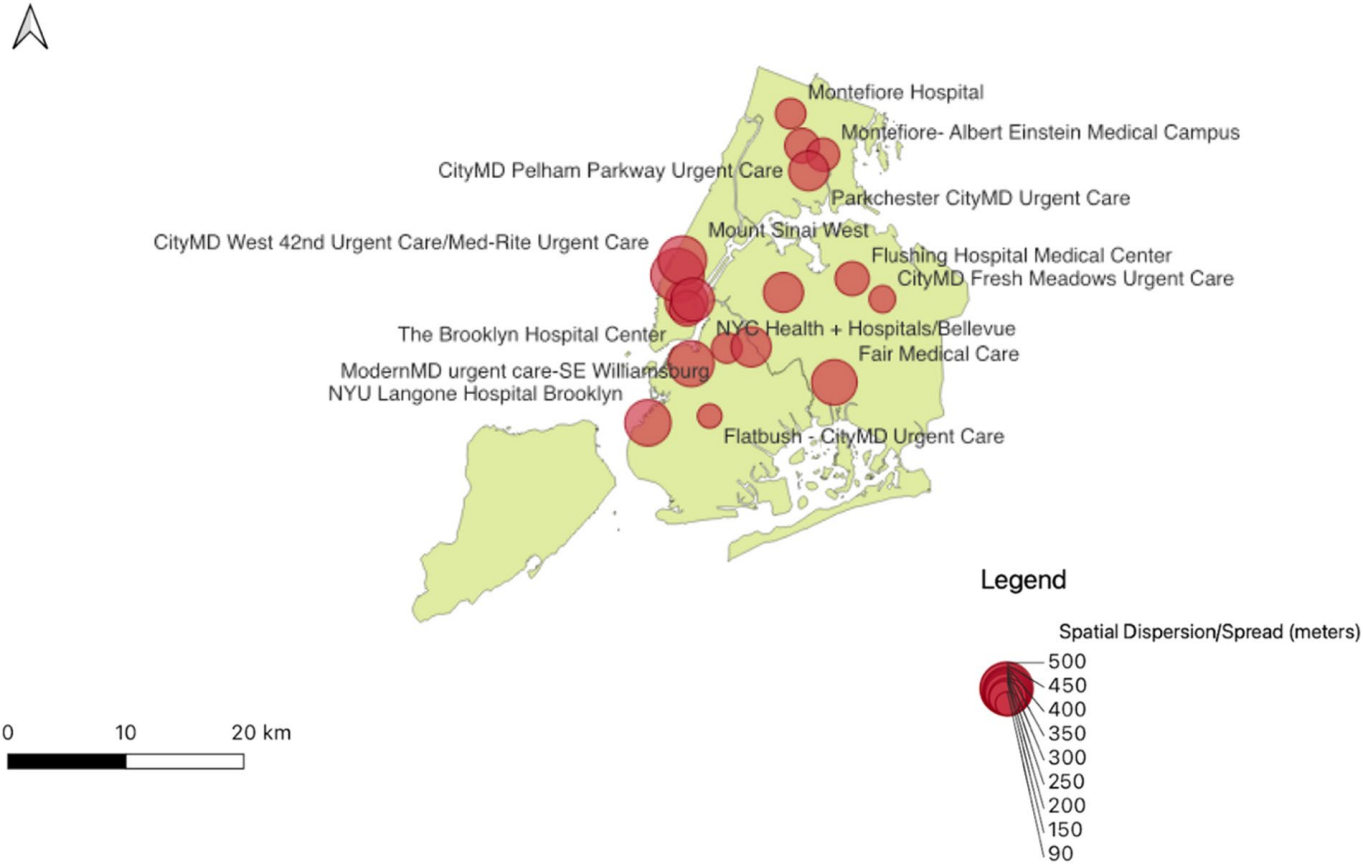
Total spatial spread walked (meters) from each HCC.

**Fig. 4:**
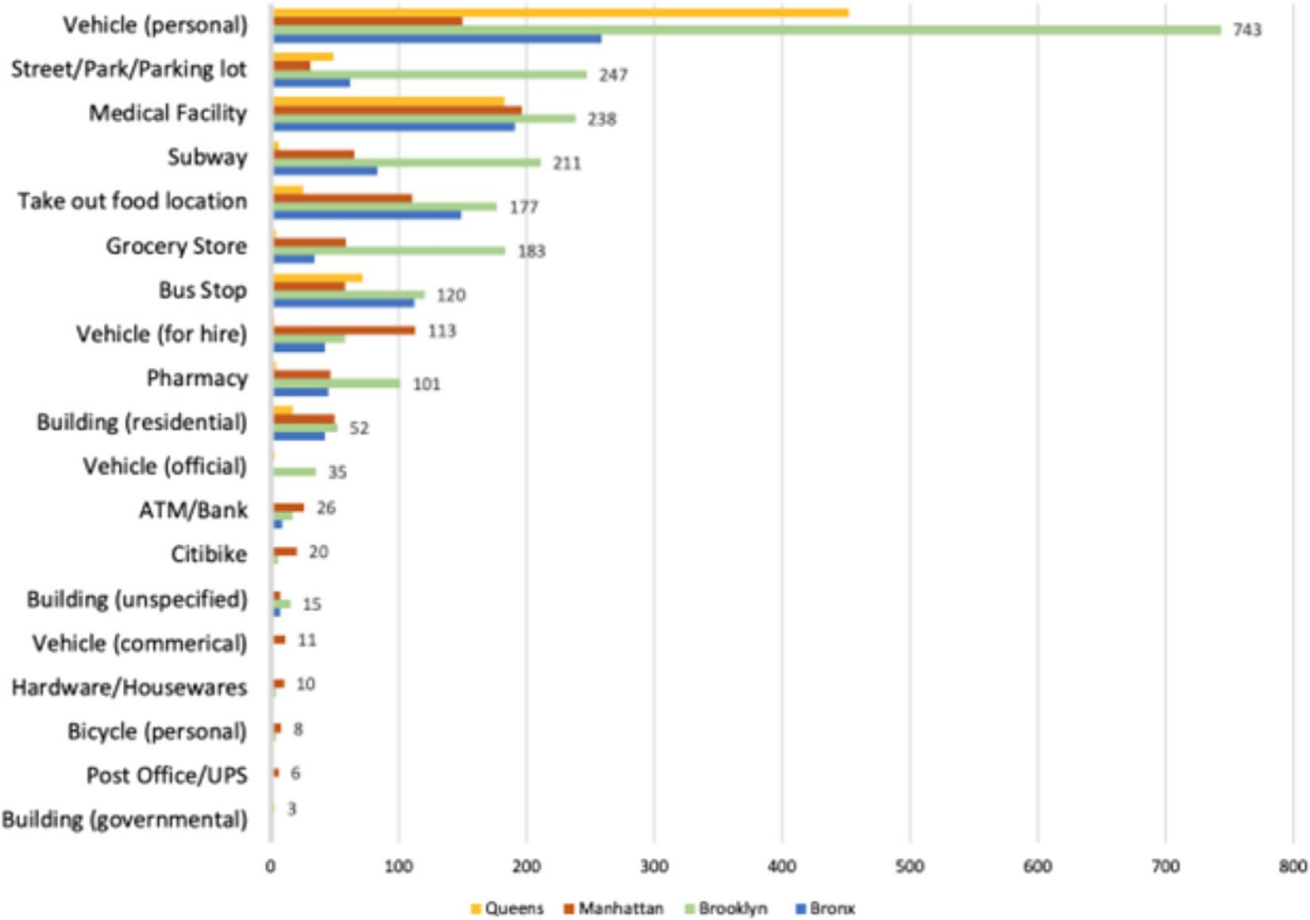
Frequency of observed final destinations of subjects exiting by borough across NYC.

Table 2 presents descriptive statistics for each HCC in the study including egress trajectory distance (length in meters) and overall spatial dispersion (egress trajectory spread) for each HCC. Average egress trajectory length (934.56 m; SD = 412.20) was observed to be somewhat shorter when emanating from urgent care clinics (N = 7; M = 851; SD; 139.07) than hospital centers (N = 11; M = 1001.00; SD=421.62), but the average *R*_*e*_ around each HCC (*R*_*e*_ = 230.99 m; SD = 111.72) was similar for both urgent care (*R*_*e*_ = 224.11; SD = 139.07) and hospital centers (*R*_*e*_ = 236.50; SD; 91.96; Fig. 3). The most common final destination for all of the subjects was their personal vehicle followed by a medical facility and take-out food location. The top 3 final destinations observed were the subjects’ personal vehicles (n = 1,604), medical facilities (n = 807), and take-out food locations (n = 461), but there was variation between NYC boroughs (Fig. 4).

**Table 2:** Characteristics of Healthcare Centers and observed egressing subjects.

Healthcare Centers	No. observed egress	Average max distance traveled (meters) (SD)	Total spatial spread (meters)
**Bronx**			
CityMD Pelham Parkway Urgent Care	443	120.08 (102.48)	158.0
Montefiore- Albert Einstein Medical Campus	346	191.99 (48.40)	143.0
Montefiore Hospital	7	105.35 (29.77)	118.0
Parkchester CityMD Urgent Care	239	137.14 (180.27)	227.0
**Brooklyn**			
Flatbush - CityMD Urgent Care	19	66.24 (62.19)	92.2
ModernMD urgent care-SE Williamsburg	188	74.21 (93.93)	120.0
NYU Langone Hospital Brooklyn	1,214	205.88 (269.05)	339.0
The Brooklyn Hospital Center	158	278.56 (159.67)	332.0
Wyckoff Heights Medical Center	631	186.58 (139.30)	233.0
**Manhattan**			
23rd St CityMD Urgent Care	334	224.08 (160.71)	276.0
CityMD West 42nd Urgent Care/Med-Rite Urgent Care	51	251.21 (428.37)	498.0
Mount Sinai Beth Israel	299	110.38 (124.81)	167.0
Mount Sinai West	45	174.10 (339.96)	383.0
NYC Health + Hospitals/Bellevue	239	209.81 (186.75)	281.0
**Queens**			
CityMD Fresh Meadows Urgent Care	303	69.58 (71.28)	99.7
Elmhurst Hospital Center Emergency Room	89	180.32 (136.46)	227.0
Fair Medical Care	42	202.87 (247.62)	322.0
Flushing Hospital Medical Center	388	89.50 (122.39)	152.0

## Technical Validation

The US Postal Service street address associated with each HCC is linked to an approximate location centroid, and thus it was known in advance that the publicly available address for each HCC would not correspond with the precise location of each HCC egress point (i.e., the specific door) under observation in the study, and thus it would be impossible to calculate accurate egress trajectory distances. To correct for this source of spatial imprecision, geographic location coordinates were captured for each HCC exit point via reverse geo-coding, utilizing QGIS and street view imagery for ortho-rectification. After joining the egress records into a geographically-explicit, relational database, the shared HCC exit-point coordinates served as the HCC origin point for calculation of the individual egress trajectory distances and made it possible to calculate the collective spatial dispersion of the individual trajectories around each HCC in the study.

**Fig. 5:**
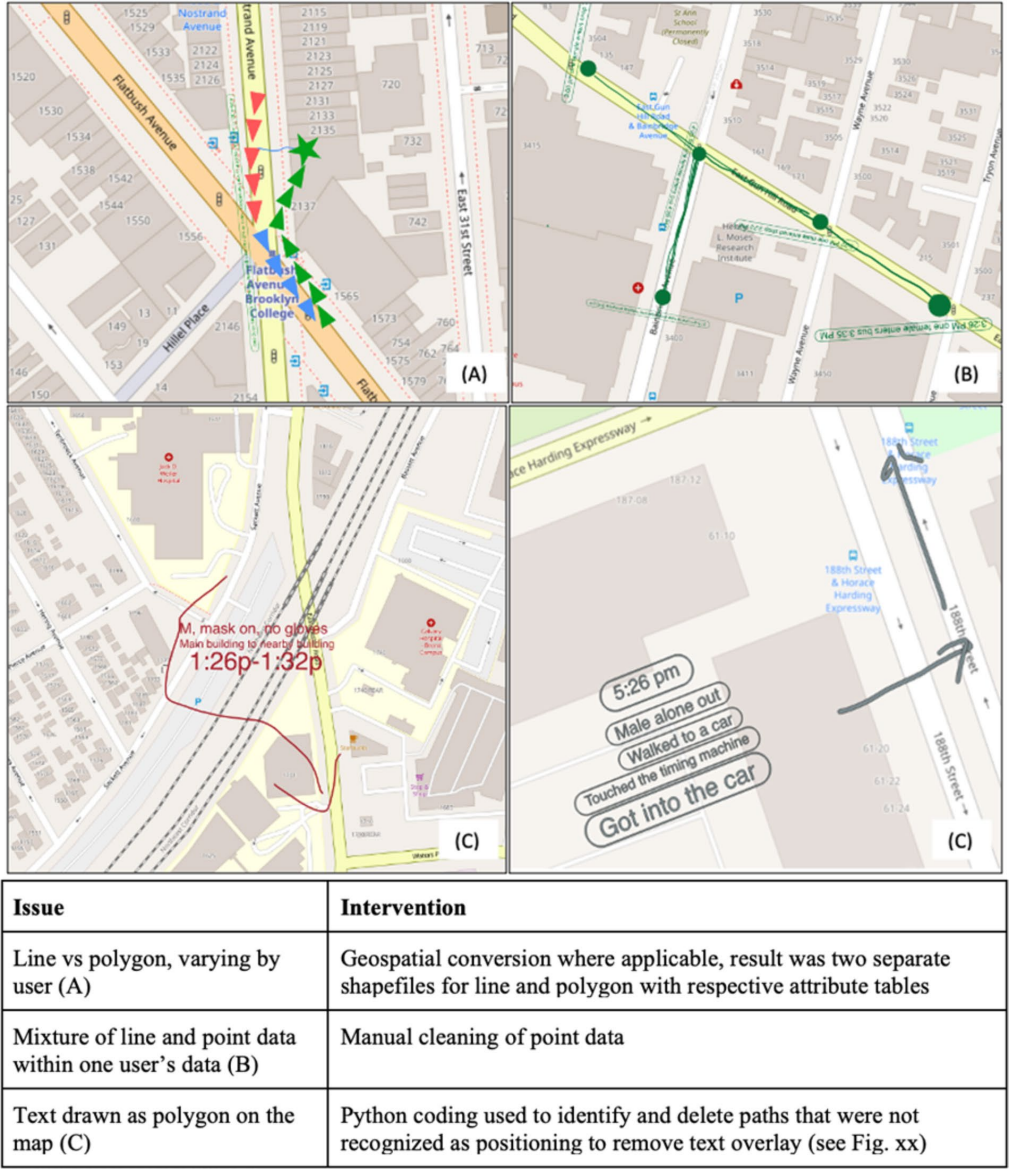
Technical Validation Issues.

Due to the urgent need to quickly dispatch observer into the field for data collectors at the outset of the COVID-19 related PAUSE order in New York City, there was insufficient time to develop and validate an interoperable data recording tool prior to deployment. As a result, prior to the geo-rectification process that linked together the data records with each HCC, some additional data cleaning needed to be performed. The amount depended upon the mobile phone OS used by each field observer (Apple versus Android), which dictate whether the smartphone records were exported to a Keyhole Markup Language (KML) or Keyhole Markup Language Zipped (KMZ) file. A QGIS bash script was used to transform the KML/KMZ files into the shapefile format. Critically only one type of spatial “geometry” (point, line, or polygon) is allowed per shapefile. Thus, some additional data cleaning and normalization was required (Fig. 5). Once normalized, all individual shapefiles were then merged into a common geo-database that compiled the egress trajectory data for all N = 18 HCCs and enabled spatial analyses.

## Usage Notes

### Data resource access and usage

Keeping the original field records produced by the study field observers is important because these raw records reflect the realities of the natural disaster scenario under which this perishable data were collected. These efforts have significant parallels in all post-disaster data collection for unprecedented events and should be considered as illustrative of the inherent challenges of doing such work and a potential roadmap for future events. The rapid response surveillance data collected as part of this NSF Rapid award provided an essential baseline for future attempts to study the way people interact with 3-D vector/environments during an extraordinary event like the global COVID19 pandemic^10,11^. By establishing an empirical snapshot of the COVID19-related vector environment surrounding these NYC healthcare facilities, communities will be better positioned to optimize public health surveillance efforts and better understand the full range of mechanisms that could be impacting the implementation of municipal ordinances moving forward^12–14^. All related project data are freely available under a CC-BY 4.0 license.

## Data Availability

No custom code was used nor is required to generate or process the datasets provided. All shape files were generated and can be opened with the opensource software QGIS or a similar GIS software platform, such as ArcGIS.
